# The dynamic monitoring of CEA in response to chemotherapy and prognosis of mCRC patients

**DOI:** 10.1186/s12885-018-4987-0

**Published:** 2018-11-07

**Authors:** Ping Yu, Mingyi Zhou, Jinglei Qu, Lingyu Fu, Xuedan Li, Ruimei Cai, Bo Jin, Yuee Teng, Jing Liu, Jing Shi, Jingdong Zhang

**Affiliations:** 1 Department of Medical Oncology, The First Hospital of China Medical University, Shenyang, 110001 Liaoning Province People’s Republic of China; 2 Department of Gynecology, Cancer Hospital of China Medical University, Liaoning Cancer Hospital & Institute, Shenyang, 110042 Liaoning Province People’s Republic of China; 3 Department of Clinical Epidemiology and Evidence Based Medicine, The First Hospital of China Medical University, Shenyang, 110001 Liaoning Province People’s Republic of China; 4 Department of Radiology, The First Hospital of China Medical University, Shenyang, 110001 Liaoning Province People’s Republic of China; 5 Department of Medical Oncology, Cancer Hospital of China Medical University, Liaoning Cancer Hospital & Institute, No.44 Xiaoheyan Road, Dadong District, Shenyang, 110042 Liaoning Province People’s Republic of China

**Keywords:** CEA change, Tumor response, Progression, Metastatic colorectal cancer

## Abstract

**Background:**

The role of carcinoembryonic antigen (CEA) change patterns in tumor response and long-term outcome is unclear. This study aimed to investigate the correlation between changes in CEA levels and tumor response as a potential prognostic model.

**Methods:**

CEA levels were determined from baseline to progression. A χ^2^ test was used to assess the correlation between CEA changes and tumor response. Univariate and multivariate COX models were used to explore the correlation of CEA changes to progression-free survival (PFS) and overall survival (OS).

**Results:**

All 114 patients were divided into five groups according to CEA change pattern (A: patients had an initial fast CEA decrease that then turned into a slow increase; B: patients had an initial slow CEA decrease that then turned to a slow increase; C: patients had a continually slow CEA increase; D: patients had a continually fast CEA increase; E: patients had an initial fast CEA decrease that then turned into a fast increase). Patients in Group A had the longest OS and PFS while Group E patients had the shortest OS. Baseline to week 12 and week 12 to week 18 change rates were consistent with tumor response and progression, respectively. An increase in CEA level by ≥2.7% from week 12 to 18 was an independent negative prognostic factor of OS.

**Conclusions:**

CEA changes mirror the tumor response to first-line chemotherapy and are associated with prognosis. CEA monitoring may be a substitute for computed tomography during the CEA stable period of treatment.

**Electronic supplementary material:**

The online version of this article (10.1186/s12885-018-4987-0) contains supplementary material, which is available to authorized users.

## Background

The 2014 European Society for Medical Oncology (ESMO) guidelines recommend the use of carcinoembryonic antigen (CEA) as a tumor biomarker for colorectal cancer. It is considered to be as accurate as computed tomography (CT) imaging in assessing the response of CRC liver metastases to chemotherapy [1].

While CT is the most popular method for evaluating the response of cancers to chemotherapy, and monitoring recurrence in patients with metastatic colorectal cancer (mCRC) [2], the use of CT exposes patients to radiation. Multiple CT scans are performed on patients with mCRC as they undergo chemotherapy and their use increases the patient’s radiation dose and even increases their risk of cancer [3]. In addition, the cost of a CT scan is $488.29 in the USA [4], £434 in the UK [5], and $260 in China, which are much higher than the cost of a single CEA test [6].

For normal gut cells, CEA is released into the lumen from the apical surface of columnar cells. As CRC cells lose their polarity, CEA begins to accumulate on the surface of cells. Because of blindness of the gland lumens and the proliferation of surrounding blood or lymphatic vessels, CEA is then released into the blood stream. As such, as the tumor size increases, the amount of CEA also increases [7]. Recently, the CEA ratio (pre-therapy/post-therapy) was demonstrated to be as accurate as CT, according to the RECIST criteria, in evaluating a patient’s response to therapy [8].

There are no reports that examine the change in CEA levels of individual mCRC patients after first-line chemotherapy. It is unknown how changes in CEA predict tumor response and disease progression and whether they are useful as a prognostic factor in determining progression-free survival (PFS) and overall survival (OS). As such, in this study we investigated whether changes in the CEA level correlated with CT scans in assessing tumor response after the initiation of first-line chemotherapy and as a prognostic factor for long-term patient outcomes.

## Methods

### Patients and clinical data

A retrospective study was carried out at the First Hospital of China Medical University in North-East China from January 2005 to December 2015. Full clinical records of 204 mCRC patients who received fluorouracil-based chemotherapy (FOLFOX, XELOX, or FOLFIRI) as first-line treatment were available for review. Thirty-three patients with normal CEA values (< 4.3 ng/mL) were excluded. Fifty-seven patients that had targeted therapy were also excluded, leaving 114 patients who were included in the study.

This retrospective study was compliant with the Declaration of Helsinki, and was approved by the Ethics Committee of the First Hospital of China Medical University (No.201581). Informed consent was obtained for each patient. And the privacy rights of patients were observed.

### Test of CEA value and assessment of CEA change

Each patient’s CEA serum concentration was measured on day one of each chemotherapy cycle. The tests were performed using an electrochemical luminescence method (Roche, MODULAR E170, Roche corresponding reagent box) at the departmental laboratory of the First Hospital of China Medical University. Changes in the concentration of CEA were assessed from baseline to progression after the initiation of chemotherapy.

### Assessment of tumor response to chemotherapy and progression by radiology

A CT scan was performed at baseline after every two cycles of chemotherapy until disease progression (PD) for each patient. All CT images were reviewed independently and retrospectively by two radiologists (Xuedan Li and Ruimei Cai) who have experience in abdominal image interpretation. The radiologists were blinded to the information of each patient’s prognosis, but were aware that the patients had been pathologically diagnosed with CRC. Complete response (CR), partial response (PR), stable disease (SD), and PD were assessed according to the RECIST criteria, version 1.1.

### Statistical analyses

The relationship between the change in CEA level and tumor response to chemotherapy, as determined by radiology according to the RECIST criteria, version 1.1, was calculated using a χ^2^ test. Kaplan-Meier survival curves stratified by CEA change at weeks 12 and 18 were plotted and compared using a log-rank test to analyze the long-term outcome of patients with different changes in their level of CEA. A Cox proportional-hazard model was used to estimate the prognostic factor of changes in CEA. Uni- and multivariate COX models were used to explore the prognostic relevance of covariates. Statistical significance was defined as *P* < 0.05. Statistical analyses were performed using SPSS, version 16.0.

## Results

### Patient characteristics

A total of 114 mCRC patients were involved in this retrospective study from January 2005 to December 2015. All patients were treated with chemotherapy alone as first-line treatment. The baseline CEA levels of all patients included in the study before treatment were all above normal values (≥4.3 ng/mL). The population of this study consisted of 69 men (60.5%) and 45 women (39.5%), and the average age was 58.9 years (Additional file 1: Table S1). For 84 patients (73.7%), the primary tumor was located in the left-side, and for 30 patients (26.3%), the primary tumor was located in the right-side. In total, 34 patients (29.8%) had surgery before commencing chemotherapy. Long-term survival data of 112 of the 114 patients (98.2%) were obtained up to December 2015, with 79 deaths (70.5%). The median follow-up time was 24.4 months with only 1.8% missing data.

### Change in CEA values at different time points

**Fig. 1:**
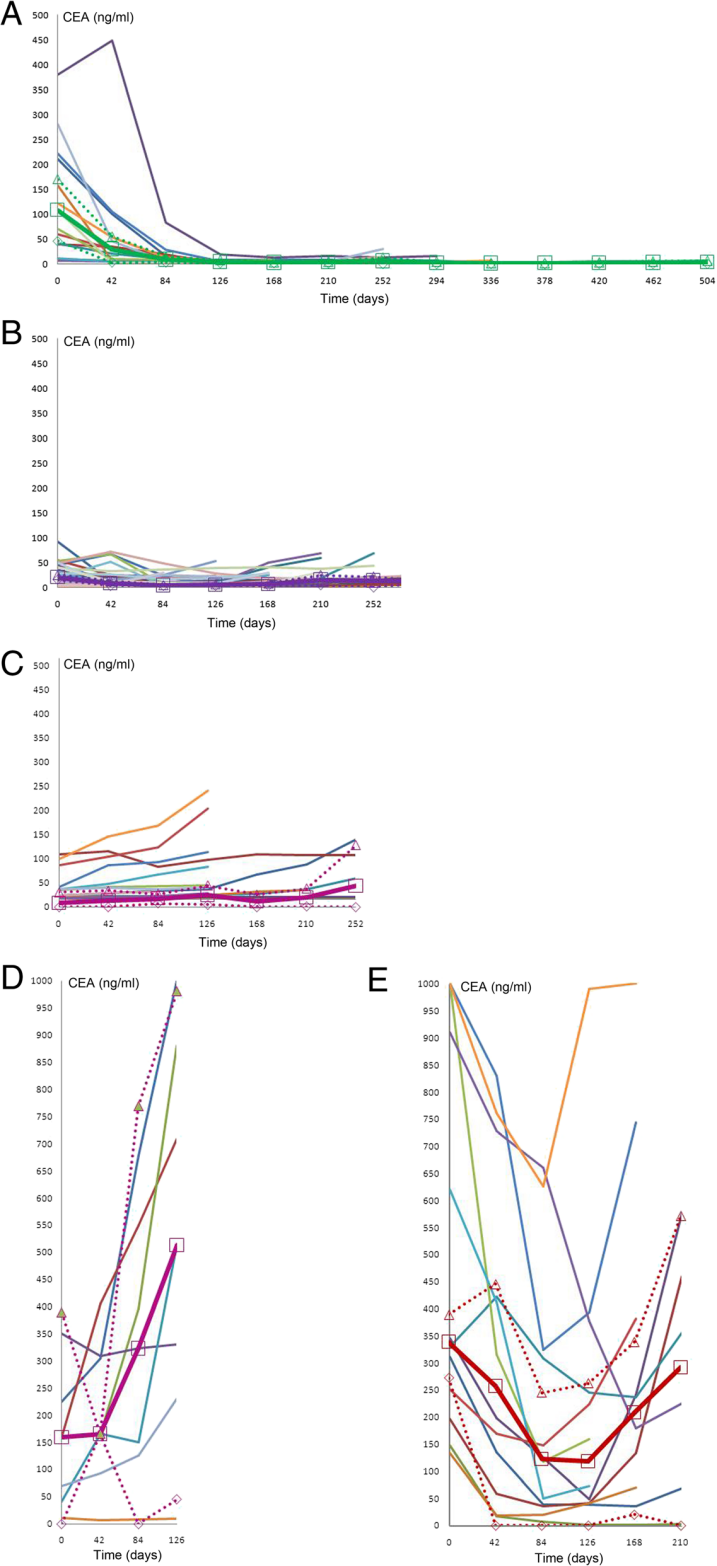
CEA change patterns of each patient at different time points. (**a**) Group A: patients had an initial fast CEA decrease that then turned into a slow increase; (**b**) Group B: patients had an initial slow CEA decrease that then turned to a slow increase; (**c**) Group C: patients had a continually slow CEA increase; (**d**) Group D: patients had a continually fast CEA increase; (**e**) Group E: patients had an initial fast CEA decrease that then turned into a fast increase

The CEA levels of 88 patients at baseline, and at weeks 6, 12, 18, and 24 after the initiation of first-line chemotherapy, are summarized in Fig. 1. Eighty-eight patients who displayed PFS and had available CEA data were stratified into five groups according to the different trends in the change of their CEA levels. Group A included patients who had an initial fast CEA decrease that then turned into a slow increase (*n* = 15, 17.0%). Group B included patients had an initial slow CEA decrease that then turned to a slow increase (*n* = 40, 45.5%). Group C included patients had a continually slow CEA increase (*n* = 14, 15.9%). Group D included patients had a continually fast CEA increase (*n* = 7, 8.0%), and Group E included patients had an initial fast CEA decrease that then turned into a fast increase (*n* = 12, 13.6%). In our study, a small number of patients were observed to have an initial transient increase in CEA level at week 6, which is consistent with the results of four previous studies^9–12^.

### Predictive accuracy of changes in CEA levels to measure tumor response

A total of 102 from the 114 patients had a measurable response by radiological evaluation, according to the RECIST criteria, at week 6 after starting chemotherapy. Objective responses (OR) includes a complete response (CR), a partial response (PR) and shrunken, but stable, disease (SD). A non-OR includes an enlarged but SD, and progressive disease (PD). At week 12, 12 patients had progressed from week 6 and were excluded, leaving 70 of 90 patients who were radiologically evaluated. At week 18, after excluding 11 patients who had progressed at week 12, 43 of 79 patients were radiologically evaluated for tumor response. A correlation between a decrease in their CEA level (≥ 50%) and radiologically confirmed OR at week 12 was observed (McNemar test, *P* = 0.678; κ = 0.33). A correlation between an increase in their CEA level and a non-OR at week 18 was also observed (McNemar test, *P* = 0.210; κ = 0.27) (Table 1).

**Table 1:** Change of CEA value at 12 and 18 weeks after the initiation of chemotherapy compared with clinical response according to RECIST criteria

	Clinical response according to RECIST criteria		
	CR + PR + shrunken SD (tumor size decreased)	Enlarged SD (tumor size increased) + PD	Total
CEA change from baseline to 12 weeks			
Decreased≥50%	19	13	32 (45.7%)
Increased or decreased < 50%	10	28	38 (54.3%)
Total	29 (41.4%)	41 (58.6%)	70 (100%)
CEA change from 12 weeks to 18 weeks			
Decreased	14	8	22 (27.9%)
Increased	8	14	22 (72.1%)
Total	22 (46.5%)	22 (53.5%)	44 (100%)

NOTE: CR, complete response; PR, partial response; SD, stable disease; PD, progressive disease

### Predictive accuracy of CEA change patterns

**Fig. 2:**
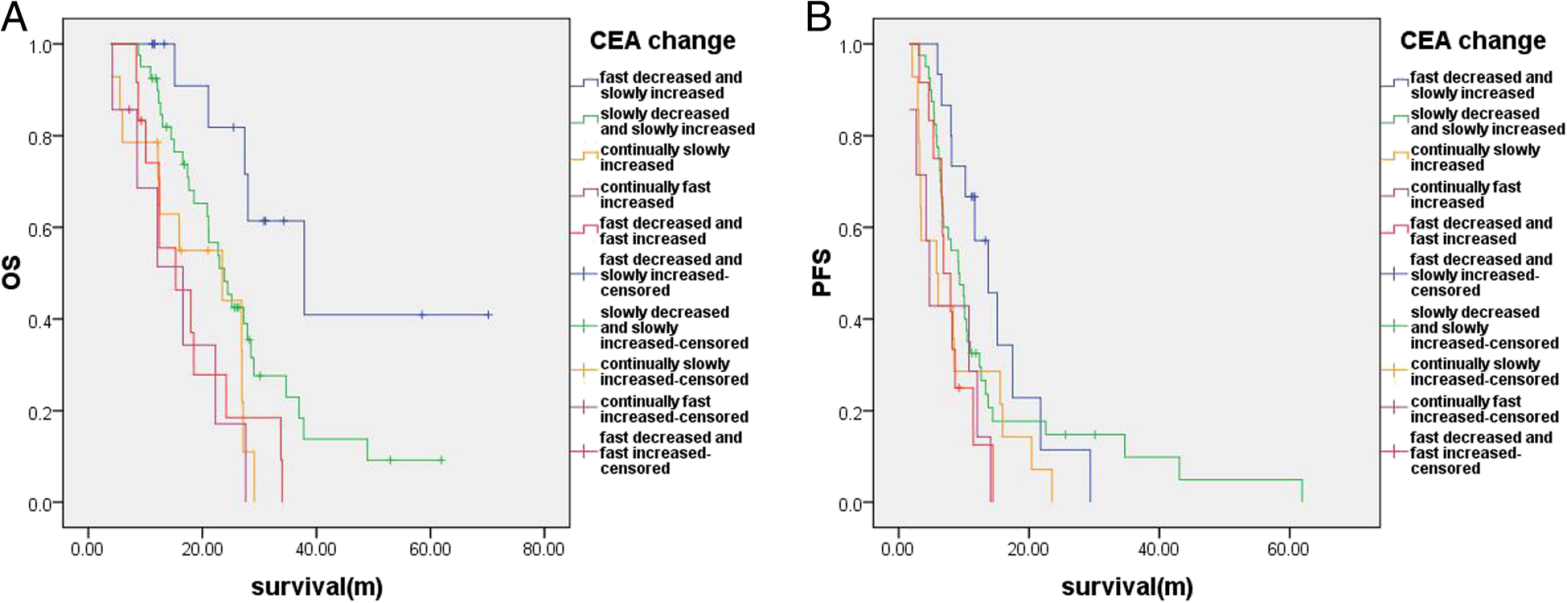
CEA change patterns affected on overall survival (**a**) and progression-free survival (**b**). Group A (Blue Curve): patients who had an initial fast CEA decrease that then turned into a slow increase; Group B (Green Curve): patients who had an initial slow CEA decrease that then turned into a slow increase; Group C (Orange Curve): patients who had a continually slow CEA increase; Group D (Purple Curve): patients who had a continually fast CEA increase; Group E (Red Curve): patients who had an initial fast CEA decrease that then turned into a fast increase

We analyzed the OS of the patients in the five groups. The median OS of the Group A patients was the longest, and the median OS of the Group E patients was the shortest (Table 2 and Fig. 2A).

**Table 2:** Comparison of median OS and PFS between four groups

	OS		PFS	
	*P* value of interval comparison	Median OS (months)	*P* value of interval comparison	Median PFS (months)
Group A vs. B	0.022	Group A: 37.9	0.229	Group A: 13.7
Group B vs. C	0.086	Group B: 23.9	0.147	Group B: 9.2
Group C vs. D	0.482	Group C: 23.5	0.429	Group C: 5.8
Group D vs. E	0.492	Group D: 16.6	0.723	Group D: 4.7
Group A vs. C	0.001		0.062	
Group B vs. D	0.013		0.164	
Group C vs. E	0.987	Group E: 15.3	0.758	Group E: 6.9
Group A vs. D	0.001		0.011	
Group B vs. E	0.021		0.179	
Group A vs. E	< 0.001		0.003	

NOTE: Bold *P*-values showed statistical significance at 0.05 level

A group: patients with fast CEA decrease and slow increase CEA, B group: patients with slow CEA decrease and slow CEA increase, C group: patients with continually slow CEA increase, D group: patients with continually fast CEA increase, E group: patients with fast CEA decrease and fast CEA increase

We also analyzed the PFS of the patients in the five groups. The median PFS of the Group A patients was the longest, and the median PFS of the Group D patients was the shortest (Table 2 and Fig. 2B).

### Best cut-off value of baseline to week 12 CEA change in predicting tumor response

**Fig. 3:**
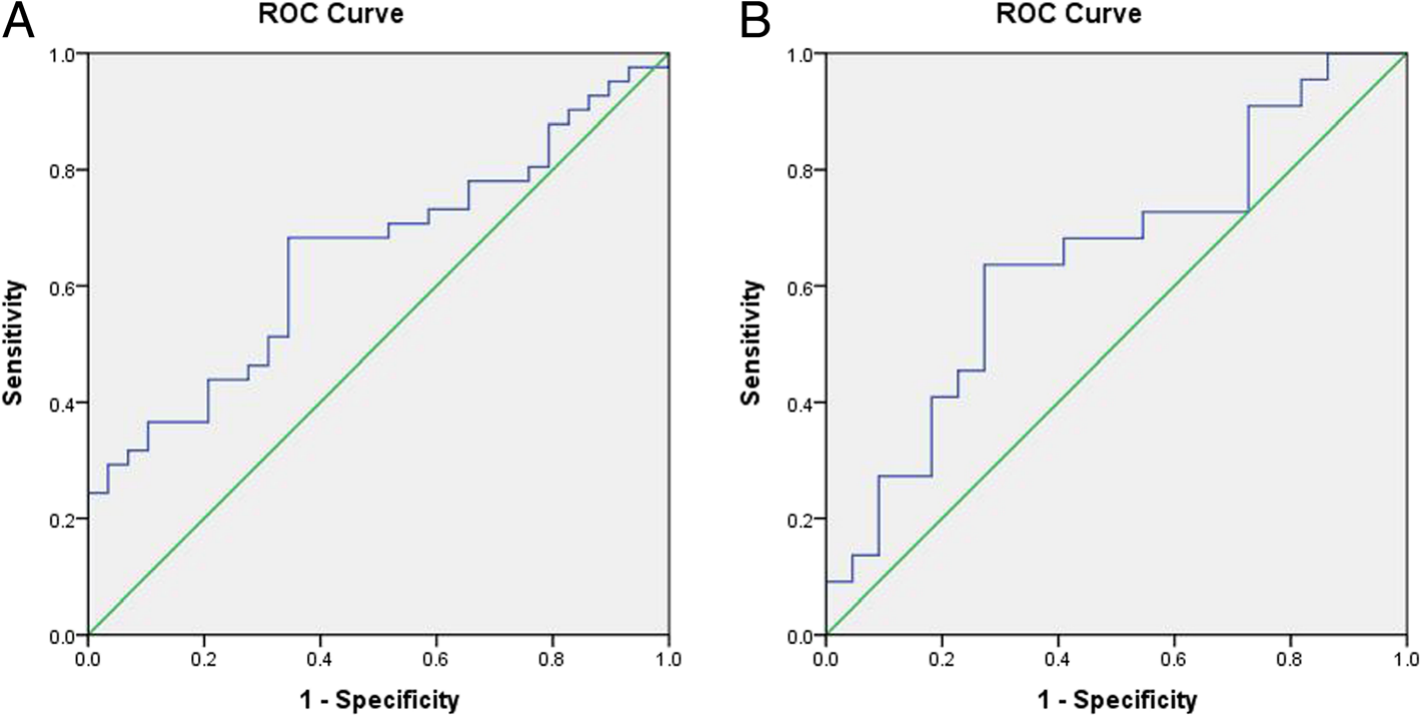
ROC curve constructed the relationship between CEA change and radiological tumor response assessed by RECIST criteria. **a**. CEA change from baseline to 12 weeks could predict radiological tumor response to chemotherapy; **b**. CEA change ratio from 12 weeks to 18 weeks could predict the radiological progression. ROC, receive operating characteristic curve

We constructed a ROC curve to determine the best cut-off value for changes in the patients’ CEA levels from baseline to week 12 for use in predicting tumor response. The dependent variable of the ROC curve was categorized by the response as determined from a radiological scan, and assessed using the RECIST criteria, on a small sample of the 75 patients at week 12. The AUC of the ROC curve was 0.65 (95%CI 0.52–0.78) (*P* = 0.033), which suggests a strong correlation in the change of CEA levels from baseline to week 12 as a variable in predicting tumor response (Fig. 3A). The best cut-off value was − 50% with a sensitivity of 68.3% and a specificity of 65.5%. This also suggested that patients with a decrease in CEA ≥ 50% from baseline to week 12 may be more sensitive to treatment.

### Best cut-off value of the change in CEA level for weeks 12–18 in predicting disease progression

We constructed a ROC curve to determine the best cut-off value for changes in the patients’ CEA levels from week 12 to 18 for predicting disease progression. The dependent variable of the ROC curve was categorized by the response as determined from a radiological scan, and assessed using the RECIST criteria, on a small sample of the 44 patients at week 18. The AUC of the ROC curve was 0.65 (95%CI 0.65–0.95) (*P* = 0.001), suggesting that a change in a patient’s CEA level from week 12 to 18 is a potential variable in predicting disease progression; however, the power was not strong (Fig. 3B). The best cut-off value was 2.7% with a sensitivity of 63.6% and a specificity of 72.7%. This result suggested that an increase in CEA ≥ 2.7% from week 12 to 18 may indicate disease progression. From survival curves of the 44 patients used to plot the ROC curve, the median OS of patients who had an increase in CEA ≥2.7% from week 12 to 18 was shorter compared with the patients who had a decrease in CEA or an increase in CEA that was less than 2.7%; 20.9 vs 27.4 months, *P* = 0.290 (Additional file 2: Fig. S1A). The median PFS of patients with an increase in CEA ≥ 2.7% from week 12 to 18 was shorter compared with the patients who had a decrease in CEA or an increase in CEA < 2.7%; 8.0 vs 12.7 months, *P* = 0.900 (Additional file 2: Fig. S1B). Furthermore, the calculated cut-off value determined from the small sample size was used to analyze the OS and PFS of all 114 patients included in the study.

**Fig. 4:**
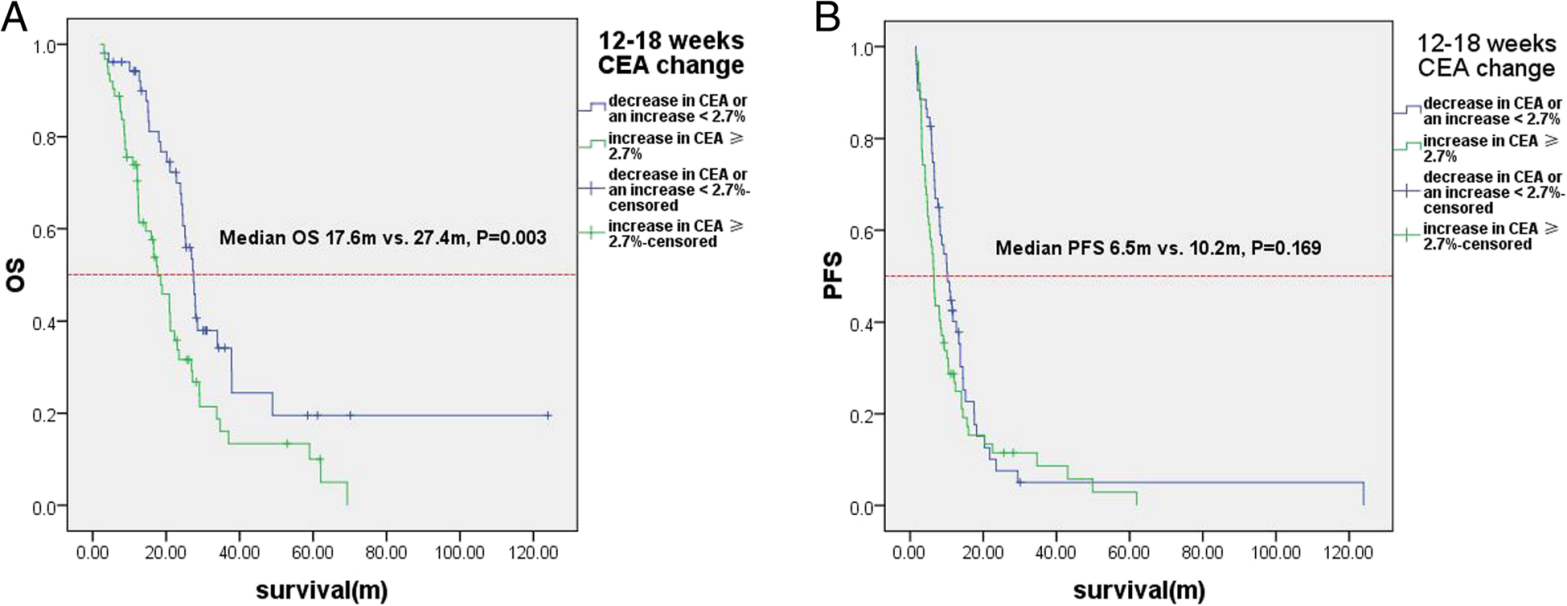
Impact of 12–18 weeks CEA change on (**a**) overall survival and (**b**) progression-free survival (validation set with total 114 patients)

From univariate analysis, the variable associated with a shorter OS was a change in CEA level from week 12 to 18 (Additional file 1: Table S2). The median OS of patients with an increase in CEA ≥ 2.7% from week 12 to 18 was significantly shorter compared with the patients who had a decrease in CEA or an increase < 2.7%; 17.6 vs. 27.4 months, *P* = 0.003 (Fig. 4A).

From univariate analysis, the variables associated with tumor progression were found to be the grade of the tumor and adjuvant chemotherapy (Additional file 1: Table S2). The median PFS of patients with an increase in CEA ≥ 2.7% from week 12 to 18 was shorter compared with the patients who had a decrease in CEA or an increase < 2.7%, but the difference was not significant; 6.5 vs. 10.2 months, *P* = 0.169 (Fig. 4B).

### Radiological response evaluation should be postponed until after week 12

During first-line treatment of mCRC, CEA screening and CT scanning are recommended at baseline and then every 6 weeks. Compared with recent surveillance of CEA screening and CT scanning during first-line treatment of mCRC, a more appropriate clinical pathway should be considered. In our study, the rate of patients with tumor progression before week 12 was 6.1%. Therefore, our results suggest that a radiological evaluation of tumor response should be performed after week 12. To confirm this hypothesis, the survival data of 125 mCRC patients from The Cancer Genome Atlas (TCGA) were analyzed. The results of the analysis showed that the rate at which patients had progressed within the first 12 weeks was 5.6%, which was consistent with our result (χ^2^ test, *P* = 0.504). As such, observing a trend in the change of a patient’s CEA levels may be of use to oncologists in recommending a radiological assessment for patients who have an increased level of CEA.

### Cost-effective analysis

According to the new monitoring model, CEA screening and CT scanning are recommended at baseline with a follow-up routine CEA screening every 6 weeks. The second CT scan should be performed at week 12 after treatment. After this, further CT scans are only warranted when there is evidence of disease progression, as determined by a change in the patient’s CEA level.

We compared the difference in costs between the two monitoring models. In China, the mean cost of a contrast CT abdominal scan and a CT lung scan is $260, whereas the mean cost of a CEA test is $18. According to our study, the median PFS in the study group was about 10 months; we used this to calculate the total fees of the two monitoring models from baseline to disease progression. In the current model, the total fee is about $3614 [($260 + $18)*13]. In our new model, the total fee would be about $1014 [($260 + $18)*3 + $18*10], which is consistent with the cost of the monitoring models used in North America and Europe.

## Discussion

Our study suggests that changes in a mCRC patient’s CEA level correlate to their tumor response to first-line chemotherapy. Patients with an early tumor response, however, did not all have long-term OS and a few patients progressed before week 12 after their chemotherapy.

Our results showed that patients in Group E had the highest baseline level of CEA that correlated to a high tumor burden, which was a predictor for a poorer long-term outcome. Even though the patients in both Groups A and E had a fast decrease in CEA after the initiation of chemotherapy, the later increase in CEA of the patients in Group A was markedly slower compared with the patients in Group E. The OS of the patients in Group A was also significantly longer when compared with the Group E patients. This suggests that early tumor response does not absolutely reflect tumor biology and that patients with a later, sharp increase in CEA concentration are likely to have a shorter survival. Moreover, the CEA level of some patients increased at week 6 after the initiation of first-line chemotherapy, which was consistent with the results of several published studies [9–12].

In 1993, Ward et al. first found that a decrease in CEA was associated with tumor response and a CEA increase was associated with disease progression; however, their study only included 33 patients, and no visible trend in the change in patients’ CEA concentrations at different time points was shown [13]. Wang et al. conducted a study of 136 mCRC patients where they tested patient CEA levels every 4 weeks and defined CEA responders as mCRC patients with a greater than 50% drop in CEA after more than 4 weeks. That study demonstrated the usefulness of CEA monitoring in determining the response to chemotherapy. While the study also examined its utility in determining the survival analysis of responders and non-responders, it did not examine the use of CEA in monitoring disease progression [14]. De Haas et al. conducted a study on 113 CRC liver metastasis patients who were treated with preoperative chemotherapy. They used a cut-off value of 20% to define a CEA responder. Their results identified a correlation between a CEA-determined response and a radiologically determined response. In their study, all patients underwent hepatic resection after a median of seven cycles of chemotherapy with the results suggesting that CEA changes defined by a cut-off value of 20% could not be used to predict PFS and OS [15].

Our results suggest that an increase in CEA ≥ 2.7% between weeks 12 and 18 predicted shorter OS. Iwanicki-Caron et al. have suggested that a CEA response, defined as a CEA slope < − 0.2, is associated with longer PFS, but no correlation between CEA kinetics and OS was assessed in their study and only those patients without a history of cancer and adjuvant chemotherapy were included. In addition, they also did not discuss the timings of CEA testing [16]. Recently, the CEA ratio (pre-therapy/post-therapy) was demonstrated as being as accurate as CT scanning, according to RECIST criteria, in evaluating therapy response. However, the authors of this study did not provide a proper clinical pathway to combining CEA testing and CT scanning in the surveillance of mCRC patients while receiving chemotherapy [8]. The results of the FIRE3 study published at the 2015 ASCO annual meeting showed that a decrease in CEA by more than 75% correlated with a longer OS; however, all the patients involved in the study were treated with targeted therapy that was combined with traditional chemotherapy. As such, their cut-off value should only be used for patients treated with anti-EGFR or anti-VEGF regimens. In the study, they also did not examine dynamic changes in the CEA concentrations of their patients [17].

Our study showed that a few patients progressed before week 12 after starting chemotherapy, which was consistent with the data calculated from TCGA. CEA screening with less reliance on radiological assessments may be an ideal cost-effective clinical pathway and should be considered for patients undergoing systematic chemotherapy. In the new monitoring model, CEA and CT scanning are recommended at baseline, followed by routine CEA testing every 6 weeks. A second CT scan would be performed at week 12 with further CT scans indicated when there is a sharp increase in the CEA concentration.

This study has some limitations. This was a retrospective study using 114 mCRC patients. The function of changes in a patient’s CEA level will be further confirmed in a prospective study with a larger number of patients treated with targeted therapy combined with chemotherapy.

In conclusion, changes in CEA levels mirror the tumor response to first-line chemotherapy and are associated with OS and PFS. CEA monitoring may be a substitute for computed tomography during the CEA stable period of treatment.

## Additional files

Additional file 1: **Table S1.** Characterization of patients. **Table S2.** Univariate and multivariate analysis of overall survival and progression-free survival. (DOCX 18 kb)

Additional file 2: **Figure S1.** Impact of 12–18 weeks CEA change on (A) overall survival and (B) progression-free survival (training set with 44 patients). (JPG 75 kb)
