# Supplementary material for: The dynamic monitoring of CEA in response to chemotherapy and prognosis of mCRC patients
Source: BMC Cancer. 2018 Nov 7;18:1076. doi: 10.1186/s12885-018-4987-0 (PMC6223053; doi:10.1186/s12885-018-4987-0)
Supplement: Supplementary file 1 — Table S1. Characterization of patients. Table S2. Univariate and multivariate analysis of overall survival and progression-free survival. (DOCX 18 kb) [file 12885_2018_4987_MOESM1_ESM.docx]

**Table S1 (published online only).** Characterization of patients.

| Characteristics | Total (N = 114) |
| --- | --- |
| Age | 58.9 ± 8.7 |
| Sex |  |
| Male | 69 (60.5%) |
| Female | 45 (39.5%) |
| Location of primary site |  |
| Left-sided tumor | 84 (73.7%) |
| Right-sided tumor | 30 (26.3%) |
| History of family |  |
| No | 90 (78.9%) |
| Yes | 24 (21.1%) |
| Grade of tumor |  |
| Poor differentiation | 19 (16.7%) |
| Moderate differentiation | 90 (78.9%) |
| Well differentiation | 5 (4.4%) |
| Prior surgery |  |
| No | 34 (29.8%) |
| Yes | 80 (70.2%) |

**Table S2 (published online early)**. Univariate and multivariate analysis of overall survival and progression-free survival

| variable | OS |  |  | PFS |  |
| --- | --- | --- | --- | --- | --- |
|  | N | UV *P* |  |  | UV *P* |
| All patients | 114 |  |  |  |  |
| Sex |  |  |  |  |  |
| Male | 69 | 0.618 |  |  | 0.427 |
| Female | 45 |  |  |  |  |
| Age |  |  |  |  |  |
| <60 years | 64 | 0.155 |  |  | 0.499 |
| ≥60 years | 50 |  |  |  |  |
| Location of primary tumor |  |  |  |  |  |
| Left-sided tumor | 84 | 0.169 |  |  | 0.925 |
| Right-sided tumor | 30 |  |  |  |  |
| Prior surgery |  |  |  |  |  |
| No | 34 | 0.663 |  |  | 0.604 |
| Yes | 80 |  |  |  |  |
| Grade of tumor |  |  |  |  |  |
| Poor differentiation | 19 | 0.172 |  |  | **0.016** |
| Moderate differentiation | 90 |  |  |  |  |
| Well differentiation | 5 |  |  |  |  |
| Baseline-12 weeks CEA change |  |  |  |  |  |
| Decreased≥50% | 56 | 0.300 |  |  | 0.895 |
| Increased or decreased <50% | 58 |  |  |  |  |
| 12-18 weeks CEA change |  |  |  |  |  |
| Decreased or increased ≤ 2.7% | 41 | **0.003** |  |  | 0.171 |
| Increased > 2.7% | 73 |  |  |  |  |
| Baseline CEA value |  |  |  |  |  |
| <200 ng/ml | 75 | 0.666 |  |  | 0.422 |
| ≥200 ng/ml | 39 |  |  |  |  |

NOTE: Bold *P*-values showed statistical significance at 0.05 level.

OS overall survival, UV univariate, MV multivariate, HR hazard ratio, CI confidence interval, CEA carcinoembryonic antigen.

Variable with univariate *P* ≤ 0.10 were entered in the Cox regression model.
