# Supplementary figures and images for: The dynamic monitoring of CEA in response to chemotherapy and prognosis of mCRC patients
Source: BMC Cancer. 2018 Nov 7;18:1076. doi: 10.1186/s12885-018-4987-0 (PMC6223053; doi:10.1186/s12885-018-4987-0)

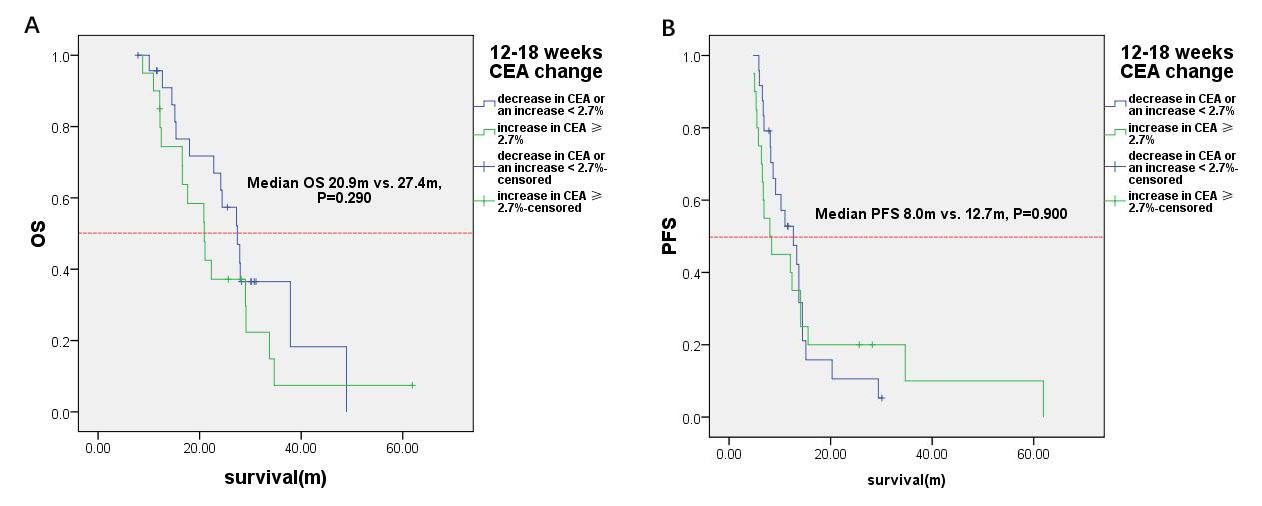

Supplement: Supplementary file 2 — Figure S1. Impact of 12–18 weeks CEA change on (A) overall survival and (B) progression-free survival (training set with 44 patients). (JPG 75 kb) [file 12885_2018_4987_MOESM2_ESM.jpg]
